# Tauroursodeoxycholic acid reduces ER stress by regulating of Akt-dependent cellular prion protein

**DOI:** 10.1038/srep39838

**Published:** 2016-12-22

**Authors:** Yeo Min Yoon, Jun Hee Lee, Seung Pil Yun, Yong-Seok Han, Chul Won Yun, Hyun Jik Lee, Hyunjin Noh, Sei-Jung Lee, Ho Jae Han, Sang Hun Lee

**Affiliations:** 1 Medical Science Research Institute, Soonchunhyang University Seoul Hospital, Seoul, Republic of Korea; 2 Department of Pharmacology and Toxicology, University of Alabama at Birmingham School of Medicine, Birmingham, AL 35294, USA; 3 Neuroregeneration and Stem Cell Programs, Institute for Cell Engineering Department of Neurology, The Johns Hopkins University School of Medicine, Baltimore, USA; 4 Department of Veterinary Physiology, College of Veterinary Medicine and Research Institute for Veterinary Science, and BK21 PLUS Creative Veterinary Research Center, Seoul National University, Seoul 151-741, Republic of Korea; 5 Department of Internal Medicine, Hyonam Kidney Laboratory, Soonchunhyang University, Seoul, Republic of Korea; 6 Departments of Biochemistry, Soonchunhyang University College of Medicine, Cheonan, 330-930, Republic of Korea

## Abstract

Although mesenchymal stem cells (MSCs) are a promising cell source for regenerative medicine, ischemia-induced endoplasmic reticulum (ER) stress induces low MSC engraftment and limits their therapeutic efficacy. To overcome this, we investigated the protective effect of tauroursodeoxycholic acid (TUDCA), a bile acid, on ER stress in MSCs *in vitro* and *in vivo*. In ER stress conditions, TUDCA treatment of MSCs reduced the activation of ER stress-associated proteins, including GRP78, PERK, eIF2α, ATF4, IRE1α, JNK, p38, and CHOP. In particular, TUDCA inhibited the dissociation between GRP78 and PERK, resulting in reduced ER stress-mediated cell death. Next, to explore the ER stress protective mechanism induced by TUDCA treatment, TUDCA-mediated cellular prion protein (PrP^C^) activation was assessed. TUDCA treatment increased PrP^C^ expression, which was regulated by Akt phosphorylation. Manganese-dependent superoxide dismutase (MnSOD) expression also increased significantly in response to signaling through the TUDCA-Akt axis. In a murine hindlimb ischemia model, TUDCA-treated MSC transplantation augmented the blood perfusion ratio, vessel formation, and transplanted cell survival more than untreated MSC transplantation did. Augmented functional recovery following MSC transplantation was blocked by PrP^C^ downregulation. This study is the first to demonstrate that TUDCA protects MSCs against ER stress via Akt-dependent PrP^C^ and Akt-MnSOD pathway.

Mesenchymal stem cells (MSCs) are promising candidates for cell-based therapies in regenerative medicine owing to their self-renewal, multidirectional differentiation, and immunomodulation potential^1^. However, application of MSCs in cell therapy has shown low therapeutic efficacy resulting from various stress conditions, including oxidative stress, inflammation, and toxic environments cause by ischemia^1^,^2^. Under these pathophysiological conditions, oxidative stress induced the production of reactive oxidative species (ROS), resulting in prolonged endoplasmic reticulum (ER) stress^3^. The ER plays a pivotal role in cell survival and homeostatic regulation. Disruption of ER homeostasis results in the accumulation of unfolded proteins and apoptosis^3^,^4^.

Cellular prion protein (PrP^C^) is a glycoprotein. Conversion into its misfolded isoform proteinase resistant protein (PrP^Sc^) causes neurodegenerative disorders, also known as prion diseases^5^,^6^. Although many efforts have been made to explore the physiological role of PrP^C^, its function is still elusive and controversial. PrP^C^ plays roles in neural precursor proliferation during developmental and adult mammalian neurogenesis^7^. In particular, PrP^C^ enhances neurogenesis and angiogenesis through proteasome activity following ischemic brain injury^8^. Moreover, PrP^C^ mediates cell adhesion via interaction with components of the extracellular matrix, such as laminin and vitronectin^9^. In hematopoietic stem cells (HSCs), PrP^C^ is expressed on long-term HSCs and regulates their self-renewal^10^. Therefore, understanding how PrP^C^ is linked to oxidative stress in ischemic conditions and what roles PrP^C^ plays in the survival of transplanted stem cells may provide insights into the protection of MSCs and development of PrP^C^-targeted therapeutics.

Tauroursodeoxycholic acid (TUDCA) is an endogenous hydrophilic tertiary bile acid produces in humans at a low level. TUDCA is approved by the U.S. Food and Drug Administration for use in biliary cirrhosis, and it is used effectively for cholestatic liver diseases^11^. Recent studies have revealed that TUDCA has an ameliorating effect on several diseases, including neurodegenerative diseases, osteoarthritis, vascular diseases, and diabetes^12^,^13^,^14^,^15^. In addition, TUDCA regulates stem cell differentiation into various lineages such as adipogenic and osteogenic lineages^16^,^17^. Mechanistic studies indicate that TUDCA attenuates ER stress, prevents unfolded protein response dysfunction, and stabilizes mitochondria^18^. However, little is known about the molecular mechanism by which TUDCA protects cells from oxidative stress. In particular, the potential for TUDCA regulation of PrP^C^ has not been investigated.

To clarify the effect of TUDCA on MSCs in ischemic conditions, we investigated whether TUDCA enhanced survival of MSCs in ischemia-induced ER stress conditions *in vitro* and *in vivo*. Results of this study reveal the mechanism by which TUDCA protects against oxidative stress by regulating Akt-dependent PrP^C^ expression.

## Results

### ER stress induced cell death in a murine ischemic model

**Figure 1:**
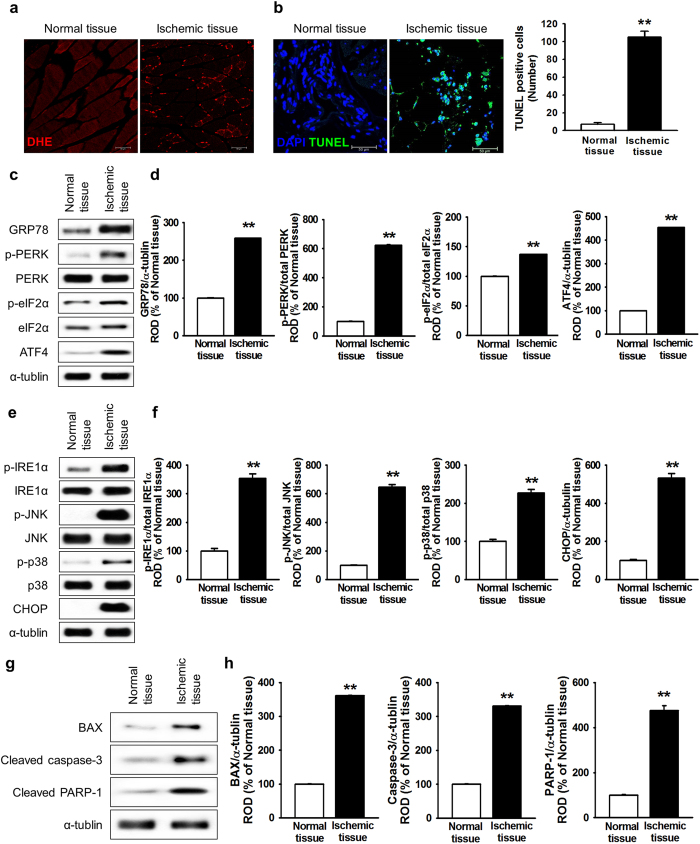
Ischemia-induced cell death via ROS-mediated ER stress. (**a**) Three days after the murine hindlimb ischemia operation, ROS levels in ischemic-injured tissues were assessed using DHE staining. (**b**) Three days after the murine hindlimb ischemia operation, apoptotic cells in ischemic-injured tissues were investigated using the TUNEL assay. The right panel represents the number of TUNEL-positive cells per high-power field. Values represent the mean ± SEM. **P < 0.01 vs. normal tissues. Scale bar = 50 μm. (**c**) Western blot analysis showing the expression of GRP78, p-PERK, PERK, p-eIF2α, eIF2α, and ATF4 in ischemic-injured tissues at postoperative day 3. (**d**) The expression levels of GRP78, p-PERK, p-eIF2α, and ATF4 were normalized to those of α-tubulin, PERK, or eIF2α, respectively. Values represent the mean ± SEM. **P < 0.01 vs. normal tissues. (**e**) Western blot analysis of p-IRE1α, IRE1α, p-JNK, JNK, p-p38, p38, and CHOP expression in ischemic-injured tissues at postoperative day 3. (**f**) The expression levels of p- IRE1α, p-JNK, p-p38, and CHOP were normalized to those of IRE1α, JNK, p38, or α-tubulin, respectively. Values represent the mean ± SEM. **P < 0.01 vs. normal tissues. (**g**) Western blot analysis showing the expression of BAX, cleaved caspase-3, and cleaved PRAP-1 in ischemic-injured tissues at postoperative day 3. (**h**) The expression levels of BAX, cleaved caspase-3, and cleaved PRAP-1 were normalized to that of α-tubulin. Values represent the mean ± SEM. **P < 0.01 vs. normal tissues. Abbreviations: DHE, dihydroethidium; TUNEL, terminal deoxynucleotidyl transferase dUTP nick end labeling; GRP78, 78-kDa glucose-regulated protein; PERK, protein kinase R-like endoplasmic reticulum kinase; eIF2α, eukaryotic initiation factor 2-alpha; ATF4, activating transcription factor 4; IRE1α, inositol-requiring enzyme 1 alpha; JNK, c-Jun N-terminal kinase; CHOP, C/EBP homologous protein; BAX, B-cell lymphoma 2-associated X protein; PARP-1, poly(ADP ribose) polymerase-1.

Ischemic-injured tissue induces ROS generation and oxidative stress, resulting in further induction of ER stress and transplanted-cell death^19^,^20^. To confirm ROS-mediated ER stress and cell death in ischemic tissue, ROS generation and cell apoptosis were assessed in ischemic-injured tissues, using a murine hindlimb ischemia model. At postoperative day 3, ROS levels were higher in ischemic-injured tissues than in normal tissues (Fig. 1a). In addition, TUNEL assay indicated that apoptotic cells were significantly higher in ischemic-injured tissues than in normal tissues (Fig. 1b and c). To investigate ROS-mediated ER stress in ischemic conditions *in vivo*, the expression and activation of ER stress-associated proteins (78-kDa glucose-regulated protein (GRP78), protein kinase R-like endoplasmic reticulum kinase (PERK), eukaryotic initiation factor 2-alpha (eIF2α), activating transcription factor 4 (ATF4), inositol-requiring protein 1 alpha (IRE1α), c-Jun N-terminal kinase (JNK), p38, and CCAAT-enhancer-binding protein homologous protein (CHOP)) in normal and ischemic-injured tissues were determined by western blot analysis (Fig. 1c–f). At postoperative day 3, ischemic-injured tissues exhibited significantly higher expression levels of ER stress markers (GRP78, ATF4, and CHOP) and phosphorylation of ER stress regulators (PERK, eIF2α, IRE1α, JNK, and p38) than those in normal tissues (Fig. 1d and f). Moreover, cell death and apoptosis-associated proteins (BCL-2-associated X protein (Bax), cleaved caspase-3, and cleaved poly(ADP ribose) polymerase-1 (PARP-1)) were significantly higher in ischemic-injured tissues than in normal tissues (Fig. 1g and h). Ischemia increased tissue death (Fig. 1g and h). These results indicate that ischemic conditions trigger ROS generation, resulting in cell apoptosis through the induction of ER stress.

### TUDCA attenuated ER stress and cell death in ischemic conditions

**Figure 2:**
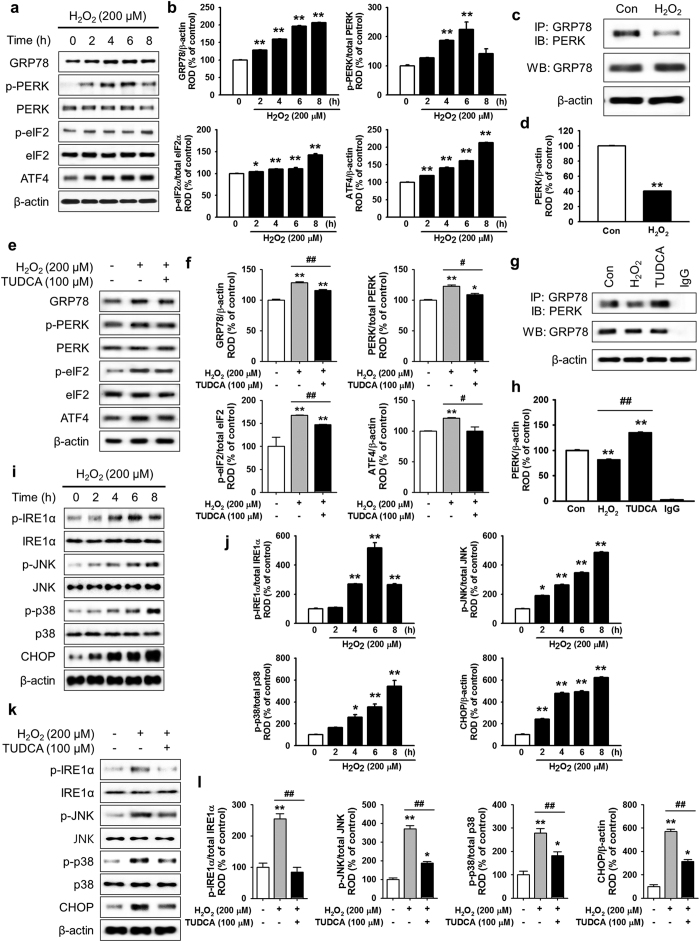
Protective effect of TUCDA against ER oxidative stress in MSCs. (**a**) Western blot of GRP78, p-PERK, PERK, p-eIF2α, eIF2α, and ATF4 expression after treatment of MSCs with H_2_O_2_ for the indicated times. (**b**) These data were normalized to β-actin, PERK, or eIF2α, respectively. Values represent the mean ± SEM. *P < 0.05 and **P < 0.01 vs. untreated MSCs. (**c**) Immunoprecipitates with anti-GRP78 were analyzed by western blot using an antibody that recognizes PERK. (**d**) The level of PERK, which binds GRP78, was normalized to that of β-actin. Values represent the mean ± SEM. **P < 0.01 vs. untreated MSCs. (**e**) Western blot of GRP78, p-PERK, PERK, p-eIF2α, eIF2α, and ATF4 expression after treatment of TUDCA-pretreated MSCs with H_2_O_2_ for 8 h. (**f**) These data were normalized to β-actin, PERK, or eIF2α, respectively. Values represent the mean ± SEM. *P < 0.05 and **P < 0.01 vs. untreated MSCs, ^#^*P* < 0.05, and ^##^*P* < 0.01 vs. treatment of MSCs with H_2_O_2_. (**g**) Immunoprecipitates with anti-GRP78 were analyzed by western blot using an antibody that recognizes PERK after treatment of TUDCA-pretreated MSCs with H_2_O_2_ (200 μM) for 8 h. (**h**) The expression level of PERK, which binds GRP78, was normalized to that of β-actin. Values represent the mean ± SEM. **P < 0.01 vs. untreated MSCs (Con), ^##^*P* < 0.01 vs. MSCs treated with H_2_O_2_. (**i**) Western blot of p-IRE1α, IRE1α, p-JNK, JNK, p-p38, p38, and CHOP expression after treatment of MSCs with H_2_O_2_ for the indicated times. (**j**) These data were normalized to IRE1α, JNK, p38, or β-actin, respectively. Values represent the mean ± SEM. *P < 0.05 and **P < 0.01 vs. untreated MSCs. (**k**) Western blot of p-IRE1α, IRE1α, p-JNK, JNK, p-p38, p38, and CHOP expression after treatment of TUDCA-pretreated MSCs with H_2_O_2_ for 8 h. (**l**) These data were normalized to IRE1α, JNK, p38, or β-actin, respectively. Values represent the mean ± SEM. *P < 0.05 and **P < 0.01 vs. untreated MSCs, ^##^*P* < 0.01 vs. treatment of MSCs with H_2_O_2_.

**Figure 3:**
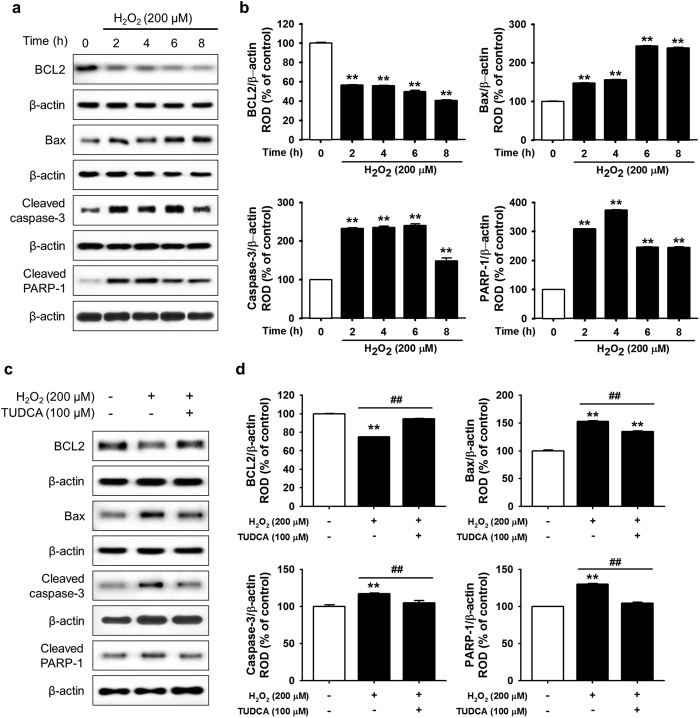
Protective effect of TUCDA against ER stress-induced apoptosis of MSCs. (**a**) Western blot analysis showing the expression of BCL-2, Bax, cleaved caspase-3, and cleaved PARP-1 after treatment of MSCs with H_2_O_2_ (200 μM) for the indicated times (0, 2, 4, 6, or 8 h). (**b**) The expression levels of BCL-2, Bax, cleaved caspase-3, and cleaved PARP-1 were normalized to that of β-actin. Values represent the mean ± SEM. **P < 0.01 vs. untreated MSCs. (**c**) Western blot analysis showing the expression of BCL-2, Bax, cleaved caspase-3, and cleaved PARP-1 after treatment of TUDCA-pretreated MSCs with H_2_O_2_ (200 μM) for 8 h. (**d**) The expression levels of BCL-2, Bax, cleaved caspase-3, and cleaved PARP-1 were normalized to that of β-actin. Values represent the mean ± SEM. **P < 0.01 vs. untreated MSCs, ^##^*P* < 0.01 vs. MSCs treated with H_2_O_2_. Abbreviations: BCL-2, B-cell lymphoma 2.

H_2_O_2_ is an ideal *in vitro* ER oxidative stress inducer. To analyze the mechanism underlying ROS-mediated ER stress in MSCs, the expression and activation of ER stress-associated proteins (GRP78, p-PERK, p-eIF2α, and ATF4) were confirmed *in vitro* after treatment with H_2_O_2_ (200 μM) for various times (0, 2, 4, 6, and 8 h). Western blot analysis showed that ER stress markers (GRP78 and ATF4) and regulators (p-PERK and p-eIF2α) were enhanced by H_2_O_2_-induced ROS (Fig. 2a and b). In particular, treatment with H_2_O_2_ significantly suppressed binding between GRP78 and PERK compared with that in untreated cells (Fig. 2c and d). To elucidate the protective effect of TUDCA on H_2_O_2_-induced ROS in MSCs, western blotting of GRP78, ATF4, p-PERK, and p-eIF2α was performed in H_2_O_2_-treated MSCs in the presence and absence of TUDCA (Fig. 2e). In ischemic conditions, treatment with TUDCA (100 μM) significantly inhibited the expression and activation of ER stress-associated proteins compared with those of untreated MSCs (Fig. 2f). Interestingly, TUDCA significantly blocked the H_2_O_2_-mediated dissociation of GRP78 and PERK (Fig. 2g and h). Moreover, the activation and expression of other ER stress-associated proteins (IRE1α, JNK, p38, and CHOP) were confirmed *in vitro* after treatment with H_2_O_2_ (200 μM) for various times (0, 2, 4, 6, and 8 h). The ER stress-mediated proteins (p-IRE1α, p-JNK, p-p38, and CHOP) were activated by H_2_O_2_-induced ROS (Fig. 2i and j). However, treatment with TUDCA (100 μM) significantly inhibited the activation of these proteins (Fig. 2k and l). To determine the mechanism of ROS-induced apoptosis, apoptosis-associated proteins (B-cell lymphoma 2 (BCL-2), Bax, cleaved caspase-3, and cleaved PARP-1) were assessed *in vitro* after treatment with H_2_O_2_ (200 μM) for various times (0, 2, 4, 6, and 8 h). The anti-apoptotic protein BCL-2 decreased and pro-apoptotic proteins Bax, cleaved caspase-3, and cleaved PARP-1 increased with ROS-mediated ER stress (Fig. 3a and b). To confirm TUDCA protection against ER stress-induced apoptosis, MSCs were pre-treated with TUDCA (100 μM), and then apoptosis-associated protein levels were measured in H_2_O_2_-induced ER stress conditions (Fig. 3c). Under ER stress, treatment with TUDCA significantly increased the expression of BCL-2 and significantly decreased the expression of Bax, cleaved caspase-3, and cleaved PARP-1, compared with that of untreated cells (Fig. 3d). These findings indicate that treatment with TUDCA protects MSCs from ER stress-induced apoptosis by inhibiting the dissociation of GRP78 and PERK and regulating the apoptosis-associated signaling pathway.

### TUDCA mediates ER stress resistance via the expression of Akt-dependent PrP^C^

**Figure 4:**
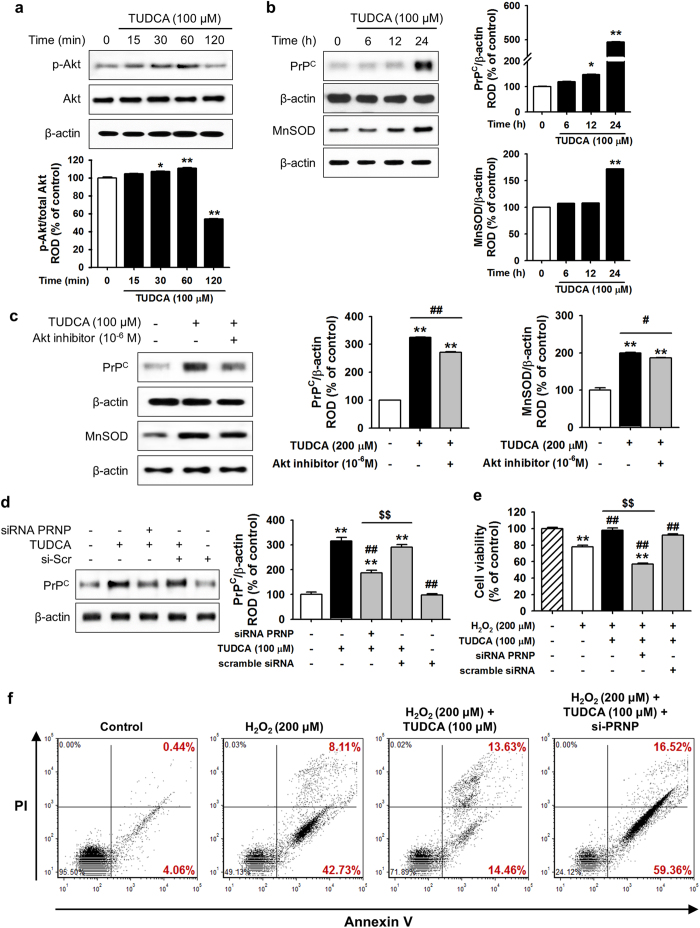
TUDCA inhibited H_2_O_2_-induced MSC apoptosis via Akt-dependent PrPC. (**a**) Western blot analysis of p-Akt expression after treatment of MSCs with TUDCA (100 μM) for the indicated times (0, 15, 30, 60, or 120 min). The lower panel shows the expression levels of p-Akt normalized to that of total Akt. Values represent the mean ± SEM. *P < 0.05 and **P < 0.01 vs. untreated MSCs. (**b**) Western blot analysis of PrP^C^ and MnSOD expression after treatment of MSCs with TUDCA (100 μM) for the indicated times (0, 6, 12, or 24 h). The right panel shows the expression levels of PrP^C^ and MnSOD normalized to that of β-actin. Values represent the mean ± SEM. *P < 0.05 and **P < 0.01 vs. untreated MSCs. (**c**) Western blot analysis of PrP^C^ and MnSOD expression after treatment of Akt inhibitor (10^−6^ M)-pretreated MSCs with TUDCA (100 μM) for 24 h. The right panel shows the expression levels of PrP^C^ and MnSOD normalized to β-actin. Values represent the mean ± SEM. **P < 0.01 vs. untreated MSCs, ^#^*P* < 0.05 and ^##^*P* < 0.01 vs. Akt inhibitor-pretreated MSCs treated with TUDCA. (**d**) Western blot analysis of PrP^C^ expression after treatment of PRNP siRNA-pretreated MSCs with TUDCA (100 μM) for 24 h. The right panel shows the expression levels of PrP^C^ normalized to that of β-actin. Values represent the mean ± SEM. **P < 0.01 vs. untreated MSCs, ^##^*P* < 0.01 vs. treatment of MSCs with TUDCA, ^$$^*P* < 0.01 vs. TUDCA-treated MSCs pretreated with scramble siRNA. (**e**) Under oxidative stress conditions (induced by treatment with H_2_O_2_), cell viabilities of untreated MSCs, TUDCA-treated MSCs, and TUDCA-treated MSCs pretreated with PRNP-specific siRNA were assessed by MTT assay. Values represent the mean ± SEM. **P < 0.01 vs. untreated MSCs in non-oxidative conditions, ^##^*P* < 0.01 vs. untreated MSCs in oxidative stress conditions, ^$$^*P* < 0.01 vs. TUDCA-treated MSCs in oxidative conditions. (**f**) Survival and apoptosis were measured using PI/Annexin V staining and flow cytometric analysis. Abbreviations: Akt, protein kinase B; MnSOD, manganese-dependent superoxide dismutase; PRNP, human PrP gene; Scr, scramble.

A previous study revealed that PrP^C^ promotes post-ischemic neuronal survival and neurogenesis in brain ischemia^8^. In considering how TUDCA protects against ER stress, we hypothesized that the TUDCA-related Akt signaling pathway regulates PrP^C^ and MnSOD. First, we analyzed the response of the Akt signaling pathway to treatment of human adipose tissue-derived MSCs with TUDCA. In a western blot analysis, treatment of MSCs with TUDCA increased phosphorylation of Akt within 30 min of treatment (Fig. 4a). To determine whether TUDCA plays a role in the regulation of Akt-mediated protein expression, the expression levels of PrP^C^ and MnSOD were investigated after treatment with TUDCA for various times (0, 6, 12, and 24 h). After 24 h of treatment, the expression levels of PrP^C^ and MnSOD dramatically increased, and expression of these proteins was suppressed by the use of an Akt inhibitor (Fig. 4b and c). To further explore whether TUDCA-related PrP^C^ expression ameliorates cell death in ROS-induced ER stress, a cell viability assay was performed under oxidative stress conditions using PRNP siRNA, which is an siRNA targeting the human PrP gene (Fig. 4d). In H_2_O_2_-induced ER stress conditions, treatment of MSCs with TUDCA significantly enhanced cell viability compared with that in the non-treatment group, while pre-treatment with PRNP siRNA significantly decreased cell viability compared with that in the TUDCA treatment and non-treatment groups (Fig. 4e). In addition, flow cytometric analysis of PI and Annexin V indicated that downregulation of PrP^C^ increased cell death in H_2_O_2_-induced ER stress conditions (Fig. 4f). These findings suggest that the protective effect of TUDCA on cells under ER stress is mediated by the Akt-PrP^C^ and MnSOD pathways, and that this mechanism may be PrP^C^-dependent.

### TUDCA-treated MSCs enhance functional recovery in murine hindlimb ischemia via PrP^C^

**Figure 5:**
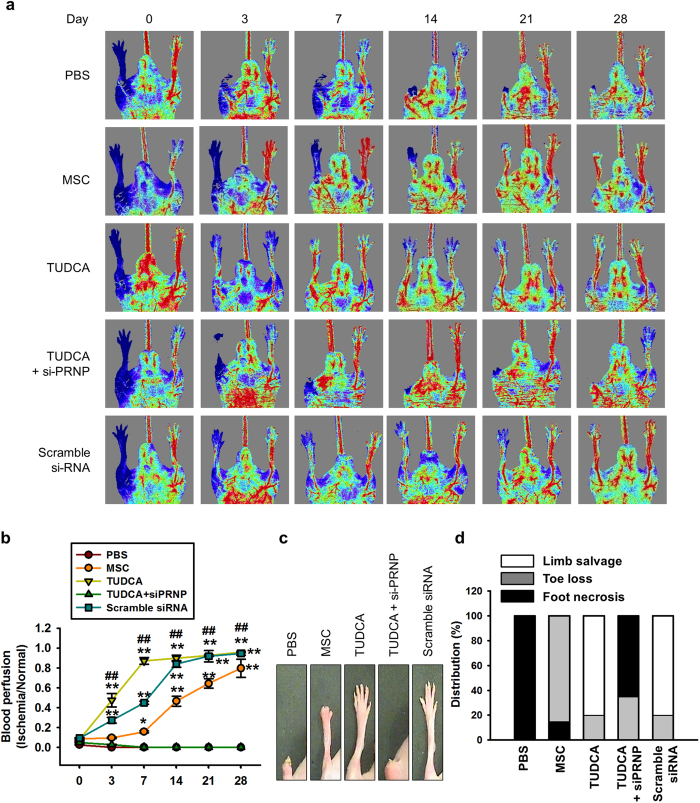
Assessment of functional recovery in a murine hindlimb ischemia model. (**a**) The murine hindlimb ischemia model was established through ligation of the proximal femoral artery and boundary vessels of 8-week-old male Balb/C nude mice. Improvements in blood perfusion were assessed by laser Doppler perfusion imaging analysis of the ischemic-injured tissues of mice injected with PBS, MSCs (MSC), TUDCA-treated MSCs (TUDCA), TUDCA-treated MSCs pretreated with PRNP-specific siRNA (TUDCA + siPRNP), and TUDCA-treated MSCs pretreated with scramble siRNA at 0 days, 3 days, 7 days, 14 days, 21 days, and 28 days postoperation. (**b**) The ratio of blood perfusion (blood flow in the left ischemic limb/blood flow in the right non-ischemic limb) was measured in each of the five groups. Values represent the mean ± SEM. *P < 0.05 and **P < 0.01 vs. PBS, ^##^*P* < 0.01 vs. TUDCA. (**c**) Representative images illustrating the different outcomes (foot necrosis, toe loss, and limb salvage) of ischemic limbs injected with five treatments at postoperative day 28. (**d**) Distribution of the outcomes in each group at postoperative day 28.

**Figure 6:**
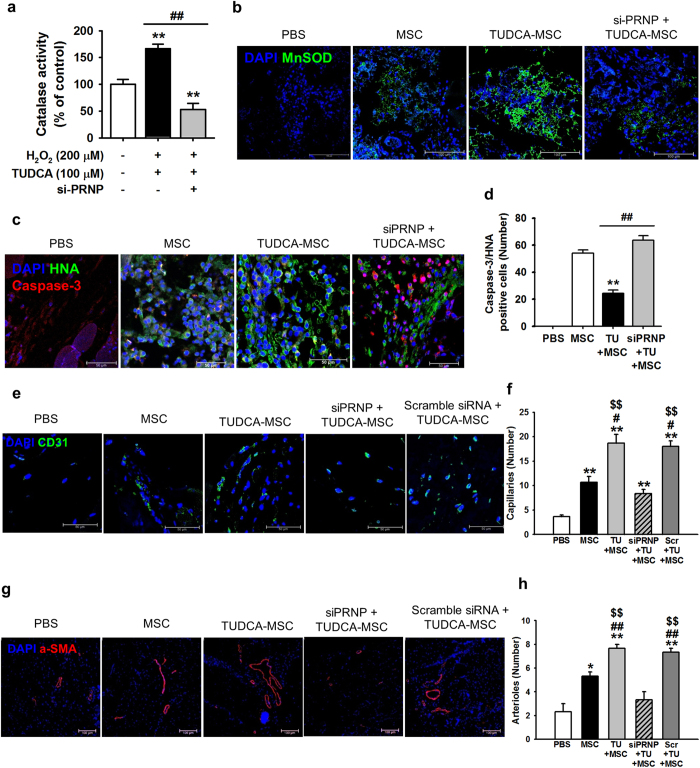
TUDCA-treated MSCs enhance functional recovery in murine hindlimb ischemia. (**a**) In oxidative stress conditions (treatment with H_2_O_2_), TUDCA-mediated catalase activity in MSCs was assessed by determining the expression levels of PrP^C^. Values represent the mean ± SEM. **P < 0.01 vs. untreated MSCs in non-oxidative condition, ^##^*P* < 0.01 vs. TUDCA-treated MSCs pretreated with PRNP-specific siRNA under oxidative stress conditions. (**b**) At postoperative day 1, immunofluorescence staining for MnSOD (green) was performed in the ischemic-injured tissues of each group. Scale bar = 100 μm. (**c**) At postoperative day 3, apoptosis of transplanted MSCs was investigated by immunofluorescence staining for HNA (green) and cleaved caspase-3 (red). Scale bar = 50 μm. (**d**) Apoptotic transplanted MSCs were quantified based on the number of HNA and PCNA double-positive cells. Values represent the mean ± SEM. **P < 0.01 vs. transplantation of MSCs, ^##^*P* < 0.01 vs. transplantation of TUDCA-treated MSCs. (**e**–**g**) At postoperative day 28, capillary density and arteriole density were assessed by immunofluorescence staining for CD31 (green; e) and α-SMA (Red; g), respectively. Scale bar = 50 μm, 100 μm. Capillary density (**f**) and arteriole density (**h**) were quantified as the number of CD31 and α-SMA positive cells, respectively. Values represent the mean ± SEM. *P < 0.05 and **P < 0.01 vs. injection of PBS, ^#^*P* < 0.05 and ^##^*P* < 0.01 vs. transplanted MSCs, ^$$^*P* < 0.01 vs. transplantation of TUDCA-treated MSCs pretreated with PRNP-specific siRNA.

To assess whether TUDCA-treated MSCs increase neovascularization *in vivo*, blood perfusion and tissue repair were investigated following transplantation of PBS, untreated MSCs (MSC), TUDCA-treated MSCs (TUDCA), TUDCA-treated MSCs pretreated with PRNP-specific siRNA (TUDCA + siPRNP), and TUDCA-treated MSCs pretreated with scramble siRNA into hindlimb ischemia mice. Blood perfusion was analyzed by LDPI at postoperative days 0, 3, 7, 14, 21, and 28 (Fig. 5a). The blood perfusion ratio was significantly greater in the TUDCA-treated MSC group than in the other groups (Fig. 5b). Moreover, transplantation of TUDCA-treated MSCs resulted in a reduction in limb loss and foot necrosis (Fig. 5c and d), and transplantation of TUDCA-treated MSCs pretreated with PRNP-specific siRNA decreased functional recovery. To evaluate the anti-oxidative effect of TUDCA-treated MSCs in ischemic conditions, the expression of MnSOD in ischemic-injured sites was assessed by immunohistochemistry after transplantation of MSCs. Prior to the *in vivo* studies, catalase activity of MSCs in ischemic conditions was assessed *in vitro* to determine whether treatment of MSCs with TUDCA augmented MnSOD activity. Treatment with TUDCA significantly increased catalase activity, but this activity was significantly decreased by PrP^C^ protein inhibition (Fig. 6a). At postoperative day 1, immunohistochemistry for MnSOD indicated that the expression of MnSOD in ischemic-injured sites was higher in TUDCA-treated transplanted MSCs than that in other groups (Fig. 6b). To confirm apoptosis of transplanted MSCs in ischemic sites, immunohistochemistry for HNA and cleaved caspase-3 was performed at postoperative day 3 (Fig. 6c). Apoptosis of transplanted MSCs was significantly lower in the TUDCA-treated group than in other groups (Fig. 6d). To investigate neovascularization by transplanted MSCs, immunohistochemistry for CD31 or α-SMA was performed at postoperative day 28 (Fig. 6e–h). Capillary density and arteriole density were significantly higher in transplanted TUDCA-treated MSCs than in the other groups (Fig. 6e–h). Inhibition of PrP^C^ protein significantly decreased the expression of MnSOD, cell survival, and vascular formation. These data indicated that TUDCA-treated MSCs facilitated vascular repair and functional recovery in ischemic-injured tissues, and that TUDCA-mediated PrP^C^ plays a pivotal role in the functionality of transplanted MSCs in these tissues.

## Discussion

Recent preclinical animal studies and clinical trials have shown that autologous and allogeneic MSCs from various sources transplanted into ischemic-injured sites localize to injured tissues^21^. However, transplanted MSCs can die in ischemic tissues, largely as a result of pathophysiological conditions such as low oxygen, high ROS levels, and inflammation^22^. This study is the first to demonstrate that TUDCA effectively protects MSCs against ER stress-related cell death and improves functional recovery of vessels through an Akt-dependent PrP^C^ signaling cascade *in vivo* and *in vitro*.

Our findings indicate that ischemic injury induces ROS generation, and that GRP78, PERK, eIF2α, and ATF4 are subsequently activated, resulting in the induction of ER stress mediated-apoptosis cascades. ER stress is caused by nutrient deprivation, hypoxic injury, redox and glycosylation reactions, and disturbances in calcium mobilization^23^. GRP78, an ER chaperone, plays a pivotal role in cell survival and death via interactions with PERK, which regulates eIF2α and the ATF4 signaling pathway^24^. In non-stress conditions, GRP78 binds to PERK, causing it to remain inactive, but ER stress causes GRP78 to dissociate from PERK, and this activation of PERK leads to eIF2α and ATF4 activation, resulting in cell death^25^. In addition, we found that TUDCA inhibits the activation of ER stress-mediated pro-apoptotic mediators, such as IRE1α, JNK, p38, and CHOP, under oxidative stress conditions. IRE1, as an ER transmembrane sensor, triggers ER stress-associated apoptosis through the decay of anti-apoptotic miRNA^26^. IRE1 also regulates the determination of cell fate via phosphorylation of JNK under ER stress conditions^27^. ER stress increases the activation of JNK and p38^28^. Furthermore, the apoptosis-related transcription factor CHOP, which promotes the expression of apoptotic genes such as cell surface death receptor 5 and BH3-only protein BIM, is regulated by the PERK-eIF2α-ATF4 pathway under ER stress conditions, resulting in the induction of apoptosis^29^. Our results show that TUDCA inhibits the dissociation of GRP78 from PERK during ER stress, preventing the activation of eIF2α and ATF4. This suggests that TUDCA protects cells from ER stress by regulating the binding of GRP78 to PERK.

Our results show that TUDCA facilitates expression of PrP^C^ in MSCs. PrP^C^ has a protective effect on cells in conditions of hypoxia, ischemia, and excitotoxicity^30^,^31^,^32^. PrP^C^ promotes long-term neuroprotection and angiogenesis in the ischemic brain^8^,^33^. PrP^C^ deficiency increases sensitivity to oxidative stress and aggravates brain ischemia^34^,^35^,^36^. In particular, downregulation of PrP^C^ increases phosphorylation of extracellular signal-regulated kinases 1/2 and reduces the activation of Akt, resulting in an increase in caspase-3 activity^36^. In addition, enhancement of Akt-mediated MnSOD expression promotes protection of MSCs against oxidative stress *in vitro* and *in vivo*^37^. Akt is a central cell signaling molecule downstream to cytokines, growth factors, and several stimulations^38^. Various stimuli induce the phosphorylation of Akt, thus activating it to regulate cellular functions such as survival, growth, proliferation, angiogenesis, metabolism, glucose uptake, migration, and invasion^38^. Under ER stress conditions, TUDCA decreases the activity of protein tyrosine phosphatase 1B, resulting in activation of the PI3K-Akt signal pathway and subsequently, the inhibition of ER stress^39^. This study, for the first time, showed that TUDCA decreased apoptosis signaling and increased cell viability under ischemic conditions via regulation of Akt-dependent PrP^C^ expression. TUDCA-induced phosphorylation of Akt enhanced the expression of both PrP^C^ and MnSOD, thus augmenting the catalase activity. However, inhibition of the Akt pathway blocked TUDCA-induced expression of PrP^C^ and MnSOD. Interestingly, knockdown of PrP^C^ did not protect against ER stress-mediated cell death. These findings indicate that TUDCA protects MSCs against ER stress through the Akt-dependent PrP^C^ signaling pathway and Akt-dependent MnSOD expression and suggest that activation of PrP^C^ is a key mechanism underlying TUDCA-mediated ER stress protection.

Finally, this study showed that TUDCA-treated MSCs enhanced functional recovery and neovascularization in a murine hindlimb ischemia model. Blood flow ratio, limb salvage, expression levels of MnSOD, transplanted cell survival, and vessel repair were all increased following transplantation of TUDCA-treated MSCs and were mediated by PrP^C^ expression. PrP^C^ knockout mice showed severe renal dysfunction and structural damage following renal ischemia/reperfusion injury^40^. Our results indicate that TUDCA-treated MSCs have enhanced bioactivities, and that transplantation of TUDCA-treated MSCs could be used in stem cell-based therapeutics for ischemic diseases. In summary, this study, for the first time, demonstrated that TUDCA protects MSCs against ROS-mediated ER stress through the Akt-dependent PrP^C^ signaling pathway, which suggests that activation of PrP^C^ is a crucial mechanism for TUDCA-mediated MSC protection. These findings also suggest that TUDCA-treated MSCs may offer new therapeutics for ischemic disease, and that understanding the regulation of PrP^C^ may provide important insights survival mechanisms of transplanted cells that will facilitate successful cell engraftment.

## Methods

### Human MSC cultures

Human adipose tissue-derived MSCs were obtained from the American Type Culture Collection (Manassas, VA, USA). MSCs were free of hepatitis B virus, hepatitis C virus, human immunodeficiency virus, and syphilis) and negative for mycoplasma. The supplier certified that the MSCs expressed MSC surface markers (CD73 and CD105) and showed adipogenic and osteogenic differentiation potential when cultured with specific differentiation media. MSCs were cultured in alpha-Minimum Essential Medium (α-MEM; Gibco BRL, Gaithersburg, MD, USA) supplemented with 10% (v/v) fetal bovine serum (FBS; Gibco BRL) and 100 U/mL penicillin/streptomycin (Gibco BRL). MSCs were incubated in a humidified incubator at 37 °C and 5% CO_2_.

### Chemical treatment of MSCs

MSCs were washed twice with phosphate buffer saline (PBS), and fresh α-MEM supplemented with 10% FBS was added. To investigate the apoptosis signaling pathway, MSCs were pretreated with TUDCA (100 μM) at 37 °C for 30 min and then treated with H_2_O_2_ (200 μM) for various times (0, 2, 4, 6, or 8 h). To assess another cell signaling pathway, MSCs were treated with an Akt inhibitor (10^−6^ M; Sigma, St. Louis, MO) for 30 min at 37 °C before treatment with TUDCA.

### Ethics statement

The Institutional Animal Care and Use Committee of Soonchunhyang University approved all surgical interventions and postoperative animal care (IACIC2013-5). Experiments were performed on 8-week-old male Balb/C nude mice (Biogenomics, Seoul, Korea) maintained in a 12-h light/dark cycle in accordance with the regulations of Soonchunhyang University, Seoul Hospital.

### Murine hindlimb ischemia model

To induce ischemia and oxidative stress and to assess neovascularization, a previously described hindlimb ischemia model was used with minor modifications^41^,^42^. Ischemia was induced by ligation of the proximal femoral artery and boundary vessels of the mice. No later than 6 h after surgery, PBS, MSCs, TUDCA-treated MSCs, and TUDCA-treated MSCs with scramble or human PrP gene (PRNP) small interfering RNA (siRNA) were injected intramuscularly into the ischemic thigh area (5 × 10^5^ cells/80 μL PBS per mouse; n = 5 for each group). Cells were injected into four ischemic sites. Blood perfusion was investigated by measuring the ratio of blood flow in the ischemic (left) limb to that in the non-ischemic (right) limb on postoperative days 0, 3, 7, 14, 21, and 28 using laser Doppler perfusion imaging (LDPI; Moor Instruments, Wilmington, DE).

### Immunohistochemistry

After 1, 3, and 28 days following operation, the ischemic thigh tissues were removed and fixed with 4% paraformaldehyde (Sigma). Each tissue sample was embedded in paraffin. Immunofluorescence staining was performed using primary antibodies against human nuclear antigen (HNA; Millipore, Billerica, MA, USA), manganese-dependent superoxide dismutase (MnSOD; Santa Cruz Biotechnology, Santa Cruz, CA, USA), cleaved caspase-3 (Santa Cruz Biotechnology), CD31 (Santa Cruz Biotechnology), and α-SMA (Santa Cruz Biotechnology) and secondary antibodies Alexa-488 and Alexa-594 (Thermo Fisher Scientific, Waltham, MA, USA). Nuclei were stained with 4′,6-diaminido-2-phenylindol (DAPI; Sigma), and immunostained samples were observed using confocal microscopy (Olympus, Tokyo, Japan).

### Dihydroethidium (DHE) staining

DHE (Sigma) was used to measure superoxide anion levels in the ischemic thigh sections. The sections were immersed in DHE (10 μM) for 30 min at 37 °C. After washing with PBS three times, samples were visualized by confocal microscopy (Olympus) at 488 nm excitation and 590 nm emission.

### TUNEL assay

The terminal deoxynucleotidyl transferase-mediated dUTP nick end labeling (TUNEL) assay was performed using a TdT Fluorescein *In Situ* Apoptosis Detection Kit (Trevigen, Inc, Gaithersburg, MD, USA). The MSCs were pre-treated with TUDCA (15 min) and then treated with H_2_O_2_ for 6 h. Next, MSCs were labeled according to the manufacturer’s instructions. Stained MSCs were visualized using a fluorescence microscope (ZEISS, Oberkochen, Germany).

### Western blot assay

The MSC homogenates (20 μg protein) were separated via 8–12% sodium dodecyl sulfate-polyacrylamide gel electrophoresis (SDS-PAGE), and the proteins were transferred to nitrocellulose. After the blots had been washed with TBST (10 mM Tris-HCl [pH 7.6], 150 mM NaCl, 0.05% Tween-20), the membranes were blocked with 5% skim milk for 1 h and incubated with the appropriate primary antibodies at the dilutions recommended by the supplier. Antibodies against GRP78, PERK, p-PERK, eIF2α, p-eIF2α, ATF4, IRE1α, p-IRE1α, JNK, p-JNK, p38, p-p38, CHOP, BCL-2, Bax, cleaved caspase-3, cleaved PARP-1, Akt, phosphor-Akt, PrP^C^, α-tubulin, and β-actin were all purchased from Santa Cruz Biotechnology. The membranes were then washed, and the primary antibodies were detected using goat anti-rabbit IgG or goat anti-mouse IgG conjugated to horseradish peroxidase (Santa Cruz Biotechnology). The bands were visualized by enhanced chemiluminescence (Amersham Pharmacia Biotech, England, UK).

### Immunoprecipitation

MSCs were lysed with a lysis buffer (1% Triton X-100 in 50 mM Tris-HCl [pH 7.4] containing 150 mM NaCl, 5 mM EDTA, 2 mM Na_3_VO_4_, 2.5 mM Na_4_PO_7_, 100 mM NaF, and protease inhibitors). Cell lysates (300 μg) were mixed with anti-GRP78 antibody (Santa Cruz Biotechnology). The samples were incubated for 4 h, mixed with Protein A/G PLUS-Agarose Immunoprecipitation Reagent (Santa Cruz Biotechnology), and then incubated for an additional 12 h. The beads were washed four times, and the bound protein was released from the beads by boiling in SDS-PAGE sample buffer for 5 min. The precipitated proteins were analyzed by western blotting with anti-PERK antibody (Santa Cruz Biotechnology).

### Inhibition of PrP^C^ expression by RNA interference

MSCs (2 × 10^5^) were seeded in 60-mm dishes and were transfected with siRNA in serum-free Opti-MEM (Gibco BRL) using Lipofectamine 2000, following the manufacturer’s instructions (Thermo Fisher Scientific). At 48 h after transfection, total protein was extracted and gene expression was determined by western blot analysis. The siRNA used to target PRNP and a scrambled sequence were synthesized by Bioneer (Daejeon, Korea).

### Cell viability assay

Subconfluent, exponentially growing MSCs were incubated in a 96-well plate with TUDCA for various times. Cell viability were determined using a modified 3-(4,5-dimethylthiazol-2-yl)-2,5-diphenyltetrazolium bromide (MTT) assay, which is based on the conversion of tetrazolium salt 3-(4,5-dimethylthiazol-2-yl)-5-(3-carboxymethoxyphenyl)-2-(4-sulfophenyl)-2-tetrazolium to formazan by mitochondrial NAD(P)H-dependent oxidoreductase enzymes. Formazan levels were quantified by measuring the absorbance at 575 nm using a microplate reader (Tecan, Männedorf, Switzerland).

### PI/Annexin V flow cytometric analysis

Apoptosis of MSCs was assessed with a Cyflow Cube 8 (Partec, Münster, Germany) after staining the cells with Annexin V-FITC and propidium iodide (PI) (De Novo Software, Los Angeles, CA). Data analysis was performed using standard FSC Express (De Novo Software, Los Angeles, CA).

### Catalase activity

Prior to enzyme activity measure, cells were plated in 100-mm tissue culture plates and grown to 70–75% confluence. Cells were washed twice in PBS and then collected and resuspended lysis buffer (1% Triton X-100 in 50 mM Tris-HCl [pH 7.4] containing 150 mM NaCl, 5 mM EDTA, 2 mM Na_3_VO_4_, 2.5 mM Na_4_PO_7_, 100 mM NaF, and protease inhibitors). Sample were incubated for 30 min on ice and centrifuged at 14000 rpm for 30 min at 4 °C. Collecting supernatant fraction was measured for protein concentrations using the Micro BCA assay (Thermo Fisher Scientific). Enzyme activity was measured as the decrease in H_2_O_2_ (200 mM) absorbance at 240 nm. The Catalase Assay Kit (Sigma-Aldrich) was used to measure the activity in milli unts of enzymatic activity per mg of protein contained in the samples (mU/mg protein).

### Statistical analysis

Results are expressed as the mean ± standard error of the mean (SEM). All of the experiments were analyzed by one-way analysis of variance (ANOVA). Some comparisons of ≥ 3 groups were made using the Bonferroni-Dunn test. A P value < 0.05 was considered statistically significant.

## Additional Information

**How to cite this article**: Yoon, Y. M. *et al*. Tauroursodeoxycholic acid reduces ER stress by regulating of Akt-dependent cellular prion protein. *Sci. Rep.* **6**, 39838; doi: 10.1038/srep39838 (2016).

**Publisher's note:** Springer Nature remains neutral with regard to jurisdictional claims in published maps and institutional affiliations.
